# IL-17 can be protective or deleterious in murine pneumococcal pneumonia

**DOI:** 10.1371/journal.ppat.1007099

**Published:** 2018-05-29

**Authors:** Neil D. Ritchie, Ryan Ritchie, Hannah K. Bayes, Tim J. Mitchell, Tom J. Evans

**Affiliations:** 1 Institute of Infection, Immunity and Inflammation, University of Glasgow, Glasgow, United Kingdom; 2 Institute of Microbiology and Infection, College of Medical and Dental Sciences University of Birmingham, Birmingham, United Kingdom; St. Jude Children's Research Hospital, UNITED STATES

## Abstract

*Streptococcus pneumoniae* is the major bacterial cause of community-acquired pneumonia, and the leading agent of childhood pneumonia deaths worldwide. Nasal colonization is an essential step prior to infection. The cytokine IL-17 protects against such colonization and vaccines that enhance IL-17 responses to pneumococcal colonization are being developed. The role of IL-17 in host defence against pneumonia is not known. To address this issue, we have utilized a murine model of pneumococcal pneumonia in which the gene for the IL-17 cytokine family receptor, *Il17ra*, has been inactivated. Using this model, we show that IL-17 produced predominantly from γδ T cells protects mice against death from the invasive TIGR4 strain (serotype 4) which expresses a relatively thin capsule. However, in pneumonia produced by two heavily encapsulated strains with low invasive potential (serotypes 3 and 6B), IL-17 significantly enhanced mortality. Neutrophil uptake and killing of the serotype 3 strain was significantly impaired compared to the serotype 4 strain and depletion of neutrophils with antibody enhanced survival of mice infected with the highly encapsulated SRL1 strain. These data strongly suggest that IL-17 mediated neutrophil recruitment to the lungs clears infection from the invasive TIGR4 strain but that lung neutrophils exacerbate disease caused by the highly encapsulated pneumococcal strains. Thus, whilst augmenting IL-17 immune responses against pneumococci may decrease nasal colonization, this may worsen outcome during pneumonia caused by some strains.

## Author summary

*Streptococcus pneumoniae* (the pneumococcus) is an important human pathogen and the commonest cause of community-acquired pneumonia. The bacterial carbohydrate capsule is a key determinant of virulence and host defence. Pneumonia follows colonization of the nasopharynx and can spread systemically. The cytokine IL-17, a key regulator of neutrophil host defence, is important in preventing colonization but its effects on pneumococcal pneumonia are not known. Here, we show that IL-17 has differing roles in host protection against pneumococcal pneumonia that depend on bacterial strain and capsule thickness. Pneumococci with thin capsules invade rapidly and IL-17 is protective; in strains with large capsules that remain in the lungs, IL-17 is detrimental. These strains evade neutrophil killing, and depletion of neutrophils improves outcome following infection. Thus, neutrophil accumulation within the lung is deleterious in pneumonia caused by heavily encapsulated pneumococcal strains. Our study provides fresh insight into the role of IL-17 in host defence against pneumococcal pneumonia, which may also be relevant in other infections caused by encapsulated bacteria that colonize before infection, such as the meningococcus and *Haemophilus influenzae*.

## Introduction

*Streptococcus pneumoniae*, or the pneumococcus, is a major cause of pneumonia, meningitis, sepsis and otitis media, with a highly significant global morbidity and mortality [1, 2]. Initially, the microbe colonizes the nasopharynx, where it may remain asymptomatically for weeks to months before eventual clearance [3]. However, following such colonization, in some individuals the bacterium can then invade other body compartments, such as the lower respiratory tract to cause pneumonia, or can spread to blood, myocardium or cerebrospinal fluid [4, 5]. The microbe has a polysaccharide capsule, which is an essential virulence factor, protecting the organism against neutrophil phagocytosis and other immune effector mechanisms [6]. The capsular serotype, of which there are over 90 different types, is an important determinant of many features of pneumococcal colonization and disease [7]. A number of studies have found stable differences between serotypes in the frequency with which they colonize the nasopharynx compared to their recovery in cases of invasive pneumococcal disease, usually blood [8]. This allows classification of pneumococci into “invasive” versus “non-invasive strains”, although serotype is not the only factor influencing invasiveness. Although less likely to cause invasive disease, when they do invade, these strains are more frequently associated with fatal outcome [9]. Those strains associated with fatal outcome are more heavily encapsulated *in vitro*. So-called “non-invasive” strains such as capsular serotype 3 are, however, commonly isolated from patients with non-bacteremic pneumonia [10] or from para-pneumonic effusions [11]. As suggested by Weinberger *et al* [9], this may reflect the difficulty with which heavily encapsulated strains may cross epithelial barriers, but by providing protection against neutrophil phagocytosis, the large capsule may allow persistence in tissues where invasion has occurred, accounting for the increased mortality produced by such strains.

Conjugate vaccines targeting the polysaccharide capsule provide excellent protection against nasopharyngeal carriage and have reduced the incidence of invasive disease [12, 13]. These vaccines work by stimulating production of opsonophagocytic antibodies that overcome the inhibitory effect of the capsule on neutrophil phagocytosis. However, some capsular serotypes not included in the vaccines have increased in prevalence following population vaccination [14–16]. This may lead to erosion of vaccine efficacy in the future, so that alternative non-capsular vaccine targets are being explored [17, 18]. Recent work has highlighted the importance of cell-mediated immunity in protecting against pneumococcal immunization [19]. In particular, cytokines of the IL-17 family secreted by cells of the Th17 lineage have been shown to accelerate clearance of pneumococci in an experimental model of nasopharyngeal colonization in mice [20], and confirmed in subsequent studies [21, 22]. Pneumococcal-specific Th17 cells have been detected in humans and mice in several studies and shown to increase in blood and lung after experimental human nasal colonization with pneumococci [23–25]. The role of γδ T-cells has also been studied in mouse models of pneumonia [26, 27].

Further studies have examined the role of IL-17 in host defence against pneumonia or other invasive disease. Mice lacking the IL-23 p19 receptor subunit showed increased bacterial load in lung and blood following pulmonary infection and reduced neutrophil influx into the lung [28]. IL-23 is known to be important in maintaining Th17 differentiation [29], suggesting that a relative lack of IL-17 might be responsible for the decreased neutrophil responses and increased bacterial load in this study; however, BAL levels of IL-17 following infection were not significantly reduced in the IL-23 p19 knockout animals [28]. Mice with prior influenza virus infection induced type I IFNs that inhibited the recruitment of neutrophils and expression of IL-17, which correlated with the host's increased susceptibility to secondary pneumococcal infection [30]. IL-17 depletion has been shown to negate the protective effect of prior colonization on experimental murine pneumococcal pneumonia [31], and exogenous IL-17 aids in lung clearance of pneumococci in an allergic inflammation model in mice [32]. Given that neutrophil recruitment is a major effect of IL-17, we hypothesized that the role of this cytokine in host defence against a particular pneumococcal strain may critically depend on the resistance of the strain to neutrophil phagocytosis, and hence on its degree of encapsulation. Here, we investigated the role of IL-17 in host defence against pneumococcal pulmonary infection using mice lacking the IL-17RA receptor subunit [33]. We show that IL-17 is protective in infection resulting from the invasive strain TIGR4 (serotype 4) with relatively thin capsule, but that absence of IL-17 actually improves outcome in pulmonary infection resulting from two heavily encapsulated strains of serotype 3 and 6B. Thus, whilst induction of a robust IL-17 response to pneumococci may be beneficial in reducing colonization, for some strains this may lead to a worsening of pneumonia and clinical outcome.

## Results

### Models of pneumonia with pneumococcal strains of different capsule thickness

**Fig 1:**
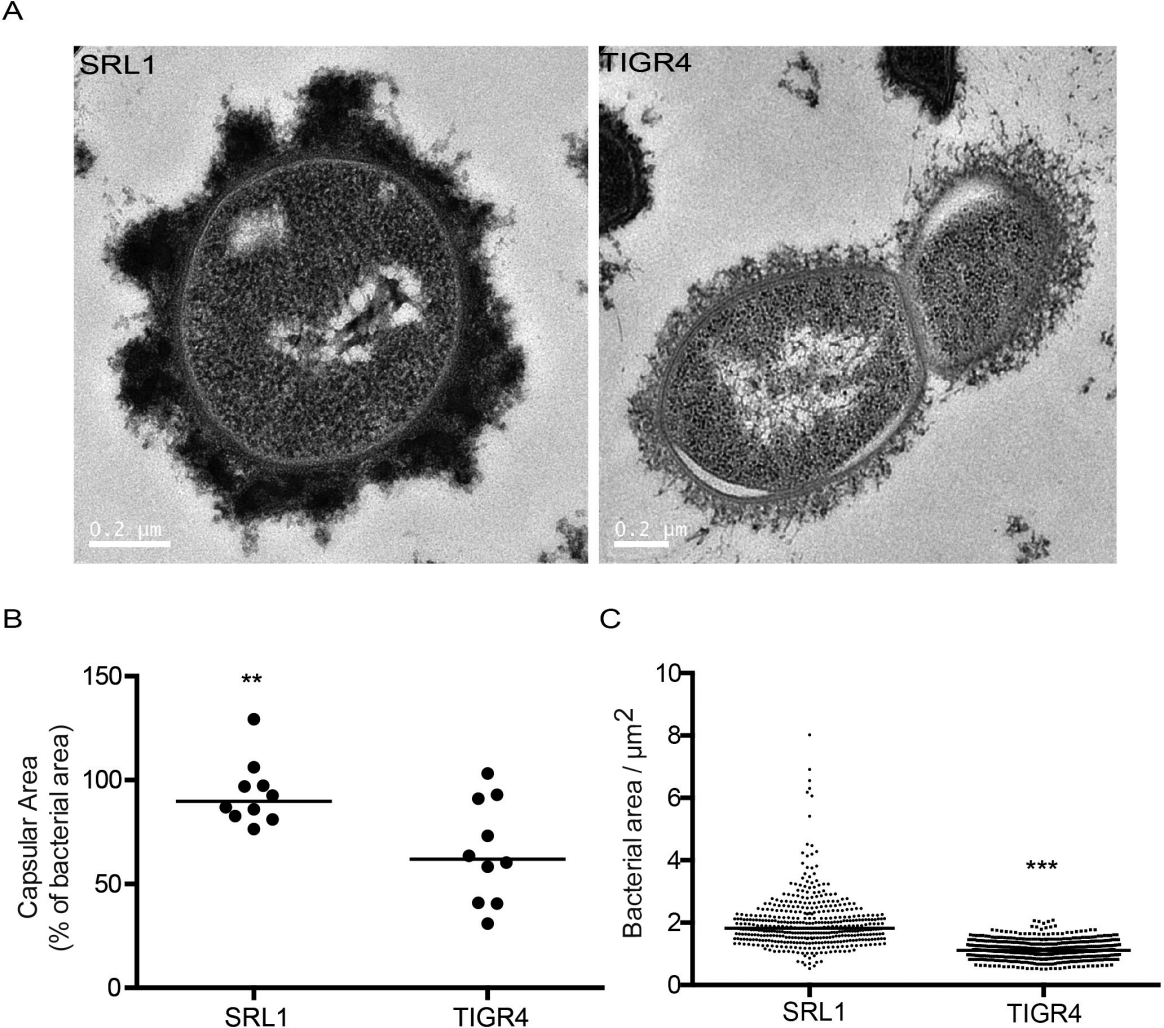
Capsular size of bacterial isolates. Bacteria were visualized using electron microscopy and fluorescence microscopy with dextran exclusion. (A) Representative transmission electron microscopy images of SRL1 and TIGR4 following fixation using ruthenium red and lysine-acetate. (B) Capsular area of SRL1 and TIGR4 as visualized by electron microscopy. (C). Area of dextran exclusion per bacterial cell. (B-C) Line shows median. (Significant difference by Mann Whitney test, ** p < 0.01, ***, p < 0.001).

*In vitro* capsular thickness of different pneumococcal strains is related to outcome of infection [9]. To explore the role of IL-17 in host defence against pneumococci with different degrees of encapsulation, we set up murine models of pulmonary infection with two strains of pneumococci differing in their capsular thickness and invasiveness potential. We chose the serotype 4 strain TIGR4 as a representative of an invasive phenotype with relatively low capsular thickness and a clinical serotype 3 isolate (SRL1) as a representative strain with high capsular thickness and low invasive potential. We confirmed these isolates differed in their capsule thickness as expected (Fig 1), with SRL1 having an average bacterial area ~60% larger than TIGR4.

**Fig 2:**
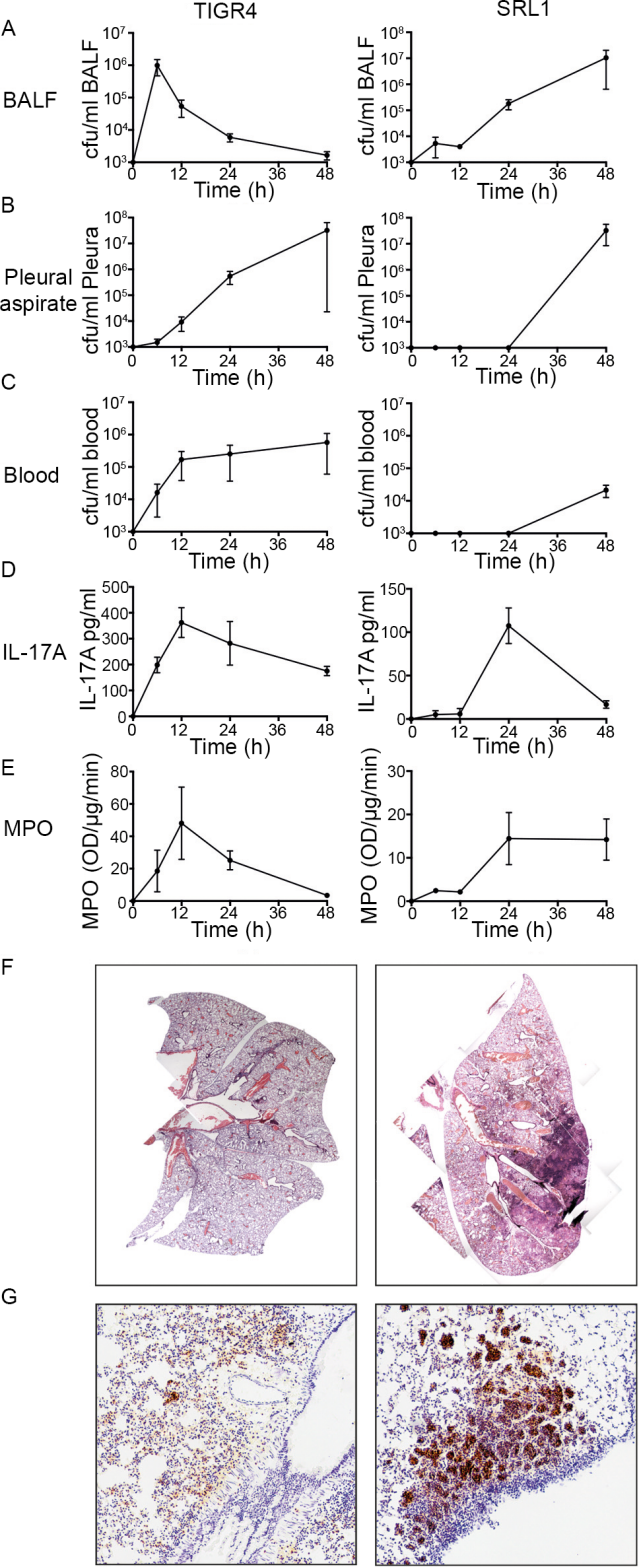
Course of infection caused by TIGR4 and SRL1. C57Bl/6 mice were infected with either TIGR4 (5 x 10^6^ cfu/mouse) or SRL1 (10^6^ cfu/mouse) by intranasal inoculation and culled at pre-determined intervals. Bacteria were enumerated in BALF (A), pleural aspirate (B) and blood (C). Concentration of IL-17A in BALF (D) and MPO in lung (E) were determined. (F) H&E tissue sections of mice infected for 48 hours at original magnification of x4. G. Immunohistochemistry using the neutrophil marker Ly-6G at an original magnification of x40. Points are means from three animals; bars show SEM. Representative of two independent experiments.

Groups of mice were infected with the different strains by intranasal inoculation under light anesthesia, resulting in aspiration of the administered inoculum into the lower airways. Animals were culled at fixed time points after infection to determine bacterial burden at different sites and associated cytokine responses. Following infection with TIGR4, there was a relatively rapid clearance of bacteria from bronchoalveolar lavage fluid (BALF) and subsequent transit to blood and the pleural space (Fig 2A–2C). This was in marked contrast to the dynamics of bacterial transit after infection with SRL1, which showed a steady accumulation in BALF and delayed transit to blood and pleural space (Fig 2A–2C). IL-17A peaked at 12 hours in BALF with TIGR4 but was delayed to 24 hours following SRL1 infection. Correspondingly, lung neutrophils showed a peak accumulation 12 hours after TIGR4 infection but a slower rise to reach a plateau after SRL1 infection (Fig 2D and 2E). Histology of the lung after infection with TIGR4 showed a patchy bronchopneumonia with peribronchial accumulations of neutrophils (Fig 2F and 2G). In contrast, after SRL1 infection, areas of dense consolidation were evident composed of larger aggregates of neutrophils (Fig 2F and 2G). Thus, taken together these data show that these different pneumococcal strains produce a different pattern of infection: TIGR4 results in a patchy pneumonia with rapid transit through the lung into blood and pleural space, while SRL1 produces a dense lobar consolidation with late transit to extra-pulmonary sites.

### Role of IL-17 in host defence against pulmonary infection with an invasive pneumococcal strain (TIGR4) of relatively low capsular thickness

**Fig 3:**
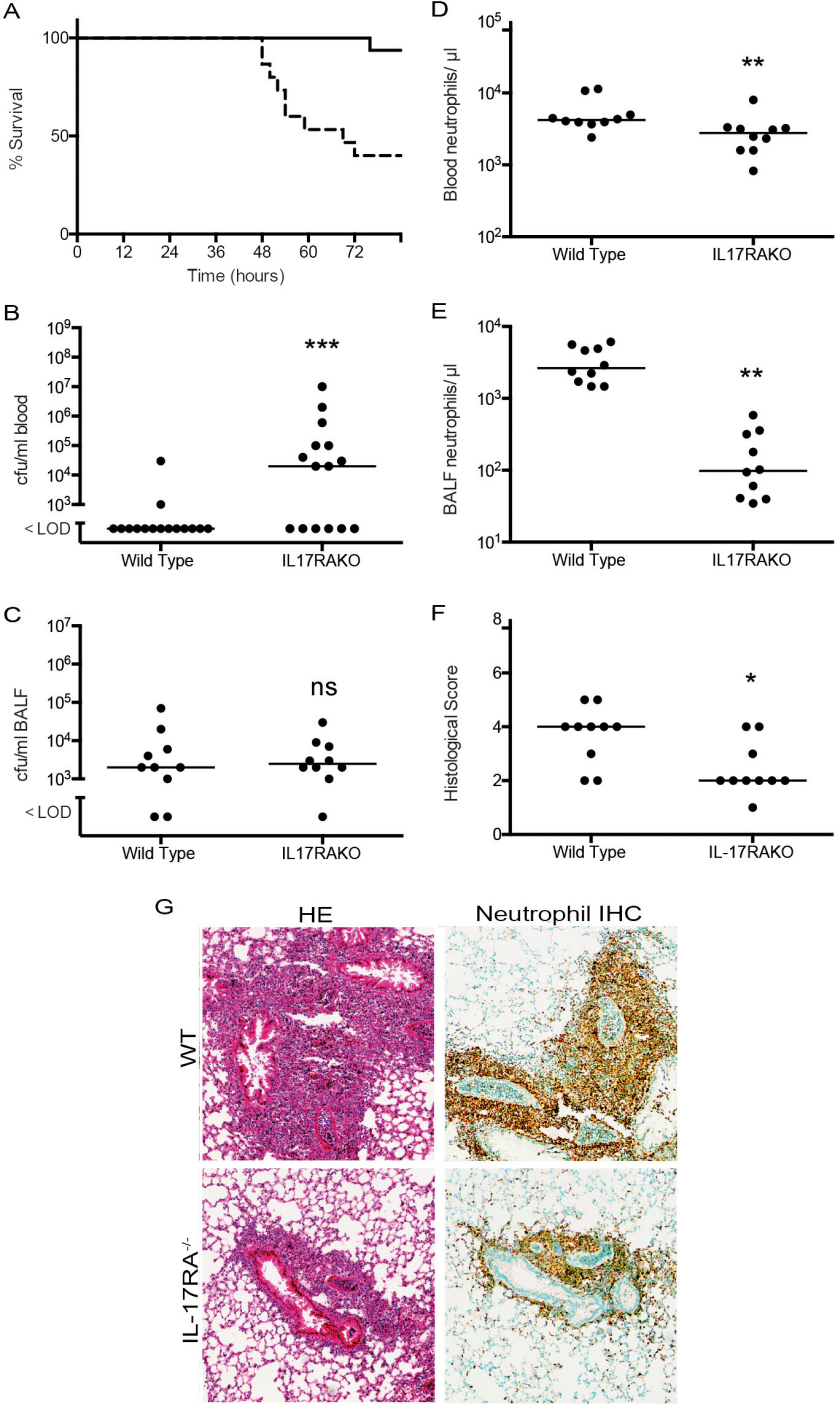
Infection of *Il17ra* KO mice with TIGR4. C57Bl/6 mice (10–16 per group) were infected with 10^6^ cfu/mouse TIGR4 by intranasal inoculation and either observed for the course of the infection or culled after 24 hours. (A) Kaplan Meier plot of survival for WT (solid line) or IL-17RA^-/-^ (dashed line), P < 0.001 (log rank test). (B) Blood and (C) BALF bacterial counts 24 h after infection. (D) blood and (E) BALF neutrophil counts 24 h after infection. (F) Lung inflammation measured by blinded assessment of hemotoxylin and eosin (HE) stained sections. (B-E), each point shows the value from an individual animal; line is median. Differences between groups were assessed by Mann Whitney test; * p <0.05, ** p< 0.01, ***, p < 0.001, ns, not significant. (G) Representative areas of inflamed lung from HE stained sections and neutrophil immunohistochemistry (Neutrophil IHC) stained lung at 24 h after infection. Original magnification x40. All data representative of two independent experiments.

To test the role of IL-17 family cytokines in this model of pneumococcal pulmonary infection, we infected groups of mice with a targeted deletion of the *Il17ra* gene, as well as with control wild type littermates. Firstly, we infected animals with the invasive pneumococcal strain TIGR4. At a dose of 10^6^ bacteria inoculated per animal, we produced a mild infection in wild type animals, with over 90% survival (Fig 3A, n = 15 animals). In the *Il17ra* knockout (KO) animals, there was a significantly increased mortality, with only 9 out of 15 animals surviving at the end of the observation period (Fig 3A, n = 15 animals). Known targets of IL-17 in lung were quantified by RT-PCR and showed large upregulation in the infected wild type animals that was virtually abolished in the infected IL-17RA knockout mice (S1 Fig).

The increased mortality in the IL-17RA knockout animals was associated with a significant increase in the recovered viable bacteria in blood at 48 hours after infection (Fig 3B). Recovered bacteria in BALF at 24 hours were at a low level and not significantly different between the WT and *Il17ra* KO animals (Fig 3C); however, blood and BALF neutrophil counts at this same time point were significantly lower in the *Il17ra* KO mice (Fig 3D and 3E). Histology of lung 24 hours after infection showed marked differences between the groups of animals. The aggregates of neutrophils surrounding airways were significantly decreased in size and number in the WT animals compared to the *Il17ra* KO mice (Fig 3F and 3G). Both sets of animals lost weight during the experiment; this was significantly less in the *Il17ra* KO animals (S2 Fig). A measure of lung edema, the wet/dry lung weight ratio, was not different between the WT and *Il17ra* KO animals (S3 Fig).

### Role of IL-17 in host defence against pulmonary infection with a highly encapsulated non-invasive pneumococcal strain (SRL1)

**Fig 4:**
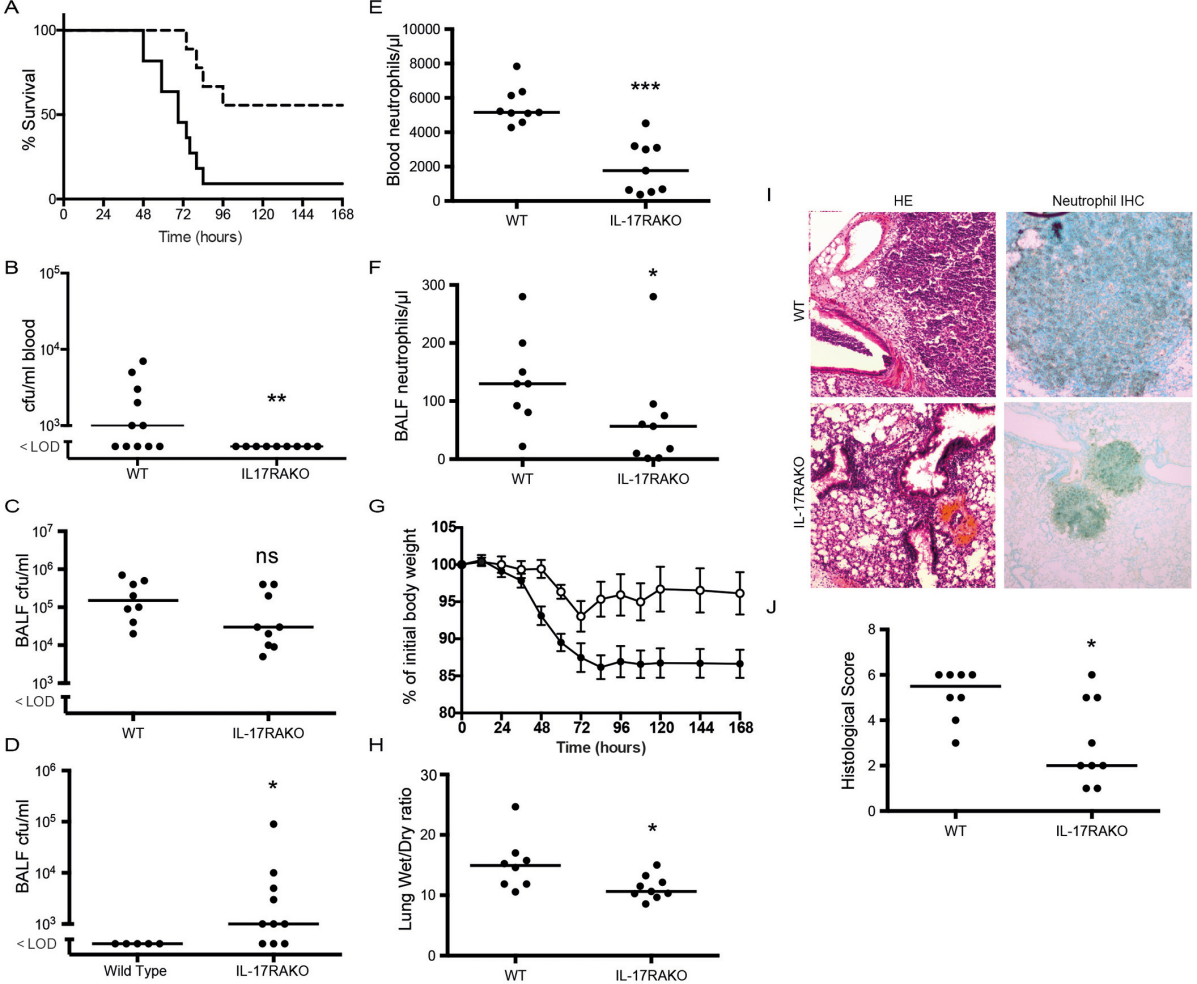
Infection of *Il17ra* KO mice with SRL1. C57Bl/6 mice (9–11 per group) were infected with 10^6^ cfu/mouse SRL1 by intranasal inoculation and either observed for the course of the infection or culled after 24 hours. A. Kaplan Meier plot of survival for WT (solid line) or IL-17RA-/- (dashed line) (P < 0.01, log rank test). (B) Blood and (C) BALF bacterial counts 48 after infection. (D) BALF bacterial counts from mice which survived infection with SRL1 (pooled from multiple experiments). (E) Blood and (F) BALF neutrophils. (G) Weight of animals following infection; open circles IL-17RA KO and closed circles WT. Each point is the mean (n = 9–11); error bar is SEM. Difference between the groups is significant, (p < 0.001, two way ANOVA). (H) lung wet/dry mass ratio at 24 hours post infection. (I) Representative areas of inflamed lung from HE stained sections and neutrophil stained lung. (J) Lung inflammation measured by blinded assessment of HE stained sections. Significance levels as in Fig 2. All data representative of two independent experiments.

The same experimental protocol was adopted to determine the course of infection of WT and *Il17ra*KO animals with the highly encapsulated non-invasive serotype 3 strain of pneumococcus, SRL1, using the same inoculum as with TIGR4 (10^6^ cfu/mouse). The *Il17ra* KO mice showed a clear and significant survival advantage following infection with this strain (Fig 4A), the reverse of the result following infection with the invasive TIGR4 strain. WT animals became bacteremic at earlier time points following infection compared to the *Il17ra* KO animals (Fig 4B). Bacterial counts within BALF from animals culled at 24 hours after infection in separate experiments were not significantly lower in *Il17ra*KO animals compared to controls (Fig 4C). However, at 7 days after infection in surviving animals from the experiments shown in Fig 4A, in 7 of the 10 surviving *Il17ra*KO animals significant numbers of bacteria could be recovered from their BALF, despite the animals remaining completely healthy and without any sign of ongoing infection (Fig 4D). No surviving WT animal had any residual bacteria within BALF.

Numbers of neutrophils in both blood and BALF in infected animals culled at 24 hours after infection were significantly lower in the *Il17ra* KO animals (Fig 4E and 4F). The weight of WT animals showed a steady decline after infection to a steady low level that was maintained over the 7 days of the experiment (Fig 4G). In contrast, *Il17ra*KO animals showed a much lower initial weight loss and a partial recovery 72 hours after infection (Fig 4G). At 24 hours after infection wet/dry lung weights were significantly higher in WT animals, consistent with increased pulmonary edema in these mice compared to the *Il17ra* KO animals (Fig 4H). Histological analysis of lung tissue in animals culled 24 hours after infection showed large areas of confluent consolidation in WT animals, largely composed of neutrophils (Fig 4I). In *Il17ra*KO animals, areas of consolidation were much smaller; these differences were significant (Fig 4I and 4J).

### Neutrophil phagocytosis and killing of TIGR4 and SRL1

**Fig 5:**
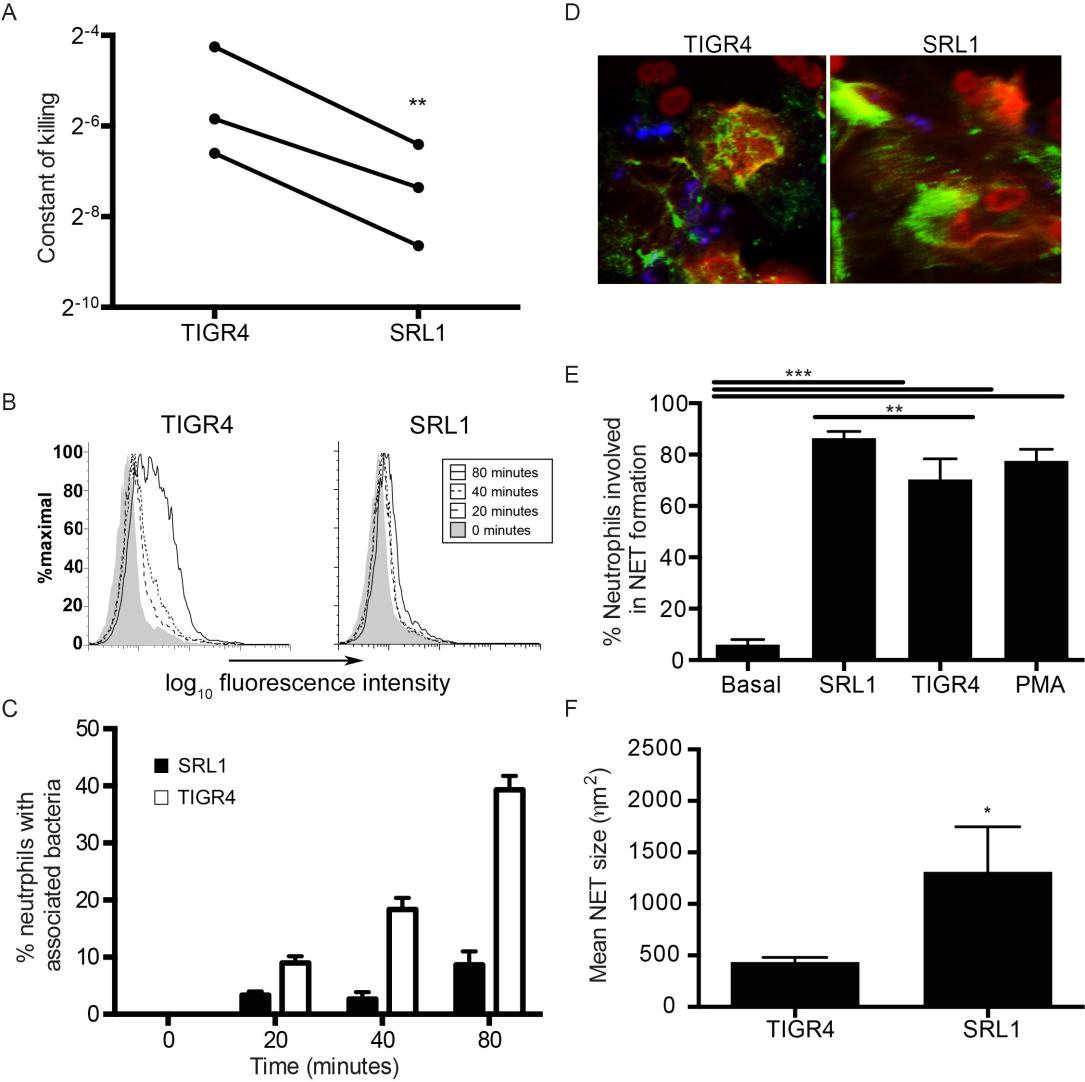
*In vitro* neutrophil phagocytosis, killing and NET formation. (A) A constant of killing was calculated using the method described by Hampton (MOI 1). The paired constants for each strain derived from 3 independent experiments are shown; differences between the two strains were significant (paired t-test, p = 0.01). (B) Fluorescent pneumococci (MOI 10) were cultured with mouse neutrophils and visualized by flow cytometry at the indicated times after bacterial addition. (C) as (B) but associated bacteria (closed bars, SRL1, open bars TIGR4) enumerated by fluorescence microscopy. Bars are means of at least 50 determinations; error bars are SEM. Differences between the strains are significant, p < 0.001, two-way ANOVA). (D-F) NET formation by neutrophils was assessed by fluorescence microscopy (MOI 10). (D) Representative confocal images of neutrophils forming NETs (blue: *S*. *pneumoniae*, green: anti-neutrophil elastase, red: sytox orange). (E) Percentage of neutrophils in NETs following incubation with indicated strains or PMA. Bars are means of at least 4 separate low power fields; error bars are SEM. Significant differences between the groups were determined by t tests with Tukey’s post hoc correction; ** p <0.01, *** p< 0.001. (F) mean NET size formed by TIGR4 and SRL1. Bars as in (E). Difference between the strains is significant, p < 0.05, t test. B-F representative of two independent experiments.

We hypothesized that the differences in outcome between WT and *Il17ra* KO mice in their response to the different pneumococcal strains were due to the effectiveness of neutrophil clearance of bacteria. To test this hypothesis, we compared the ability of neutrophils *in vitro* to take up and kill the two pneumococcal strains. Using a killing assay as described in the Methods in whole complement containing serum, we measured the rate constant of neutrophil clearance of the different strains (Fig 5A). This was consistently significantly lower for clearance of SRL1 compared to TIGR4. Direct measures of phagocytosis by two different methods also showed a marked reduction in neutrophil uptake of SRL1 compared to TIGR4 (Fig 5B and 5C). Pneumococci induce neutrophils to form neutrophil extracellular traps (NETs). Both TIGR4 and SRL1 induced NET formation (Fig 5D and 5E). SRL1 induced more traps than TIGR4 and these were significantly larger (Fig 5F and 5G). Pneumococci are reported to be relatively resistant to NET mediated killing, but NET formation has been reported to enhance development of pneumococcal otitis media [34]. The enhanced NET formation produced by SRL1 may thus be important in retaining this microbe in lung; this is considered further in the Discussion.

### Neutrophil depletion increases survival after SRL1 infection

Levels of neutrophils in the lungs of SRL1 infected animals are sustained (Fig 2) and are less able to kill this strain of pneumococcus compared to TIGR4 (Fig 5). Given that a major effect of IL17 is to enhance neutrophil production and mobilization, we hypothesized that the differing effects of IL17 on outcome from infection with these two strains manifest in Figs 3 and 4 could be due to neutrophils enhancing survival in infection with the invasive TIGR4 serotype but increasing mortality in infection with the non-invasive SRL1 strain. To test this directly, we depleted neutrophils in mice using a cytotoxic highly specific antibody directed against the neutrophil marker Ly6G, that does not cross react with other leucocytes [35]; animals were then infected with the different pneumococcal strains.

**Fig 6:**
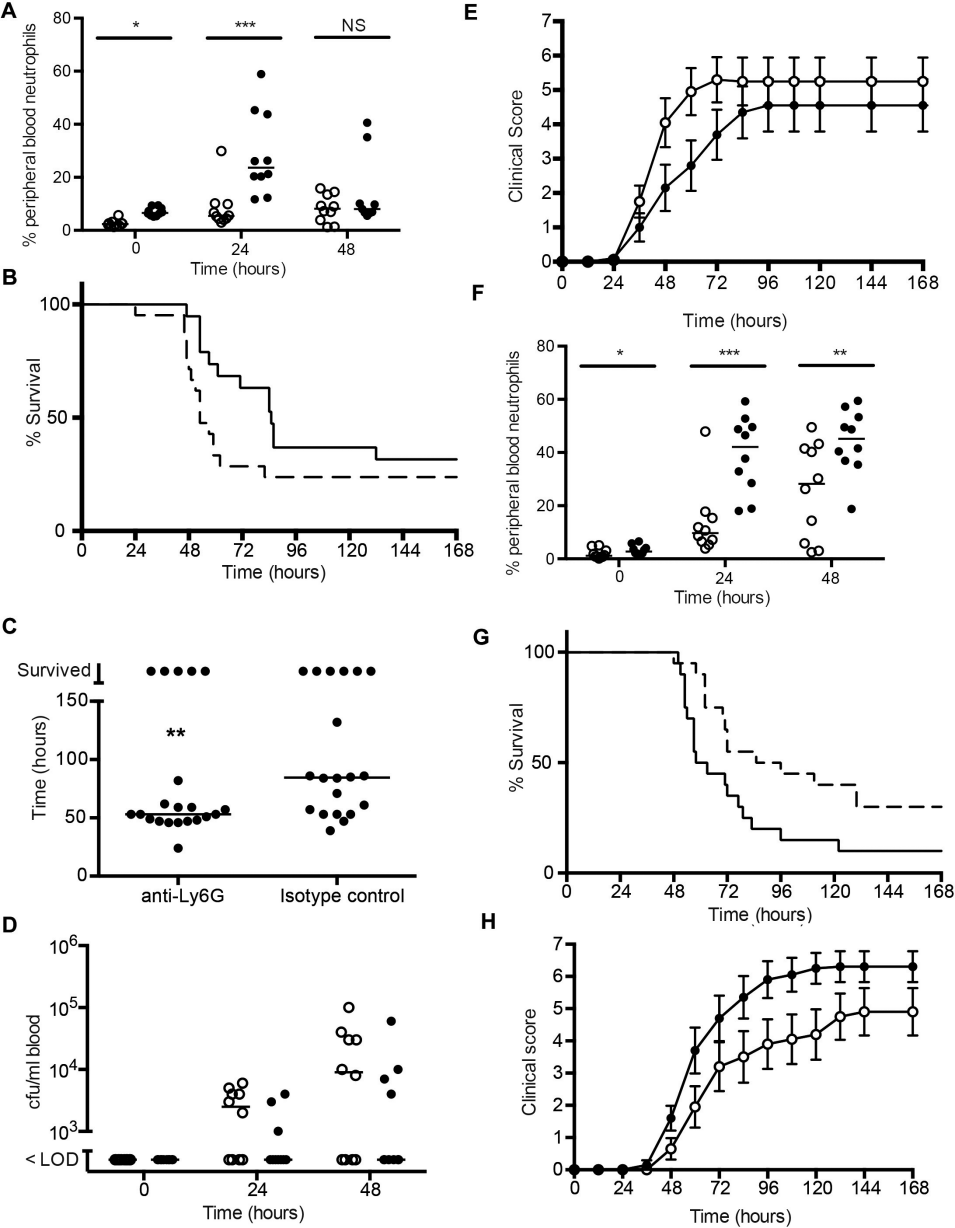
Infection with TIGR4 or SRL1 following depletion of neutrophils. C57Bl/6 mice (20 per group) were infected with 10^6^ cfu/mouse of TIGR4 (A-D) or SRL1 (E-F) by intranasal inoculation 24 hours after intraperitoneal inoculation of 500 μg antibody 1A8 (anti-Ly6G) or isotype matched control and observed for the course of the infection. (A) peripheral blood neutrophil counts following treatment with isotype control (filled circles) or anti-Ly6G (open circles) at indicated times following infection. Differences (Mann Whitney test) were not significant (NS) or significant (*, p < 0.05, ***, p < 0.001) (B) Isotype control treated mice (solid line) and neutrophil depleted mice (dashed line) had similar survival (A, P = 0.09, log rank test). (C) Median survival time among neutrophil depleted mice; each point is an individual animal, line is median. Differences are significant (**, Mann Whitney test, p = 0.01). (D) bacteremia and (E) clinical score between isotype treated mice (solid circles) and neutrophil depleted mice (open circles). Differences are not significant. (F) as (A) following infection with SRL1 (G) Outcome following SRL1 infection. Isotype control treated mice (solid line) had impaired survival compared to neutrophil depleted mice (dashed line) (p = 0.02, log rank test). (H) Clinical score in neutrophil depleted mice (open circles) compared to isotype treated mice (solid circles). Differences between the groups are significant (p = 0.01, 2-way ANOVA corrected for repeated measures).

In animals infected with TIGR4, the anti-Ly6G treatment produced a significant reduction in peripheral neutrophil counts following infection, largely abrogating the rise seen in the control isotype treated mice (Fig 6A). Survival following infection showed a trend towards decreased survival in the anti-Ly6G treated group although this did not reach statistical significance (Fig 6B). However, the time to death in the two groups showed a statistically shorter interval in the anti-Ly6G treated mice (Fig 6C). Bacteremia also showed a trend towards earlier onset and increased levels in the anti-Ly6G animals, and clinical scores were also worse in this group; however, neither of these differences reached statistical significance (Fig 6D and 6E). In addition, there was a trend towards greater weight loss following infection in the neutrophil-depleted animals.

In animals infected with the SRL1 strain, the anti-Ly6G treatment produced a sustained reduction in peripheral neutrophils (Fig 6F). Compared to the isotype-treated controls, the mice that received the anti-Ly6G antibody had a clear and significant survival advantage (Fig 6G). They also had a statistically significant reduction in clinical score after infection (Fig 6H) and a trend towards decreased weight loss after infection.

**Fig 7:**
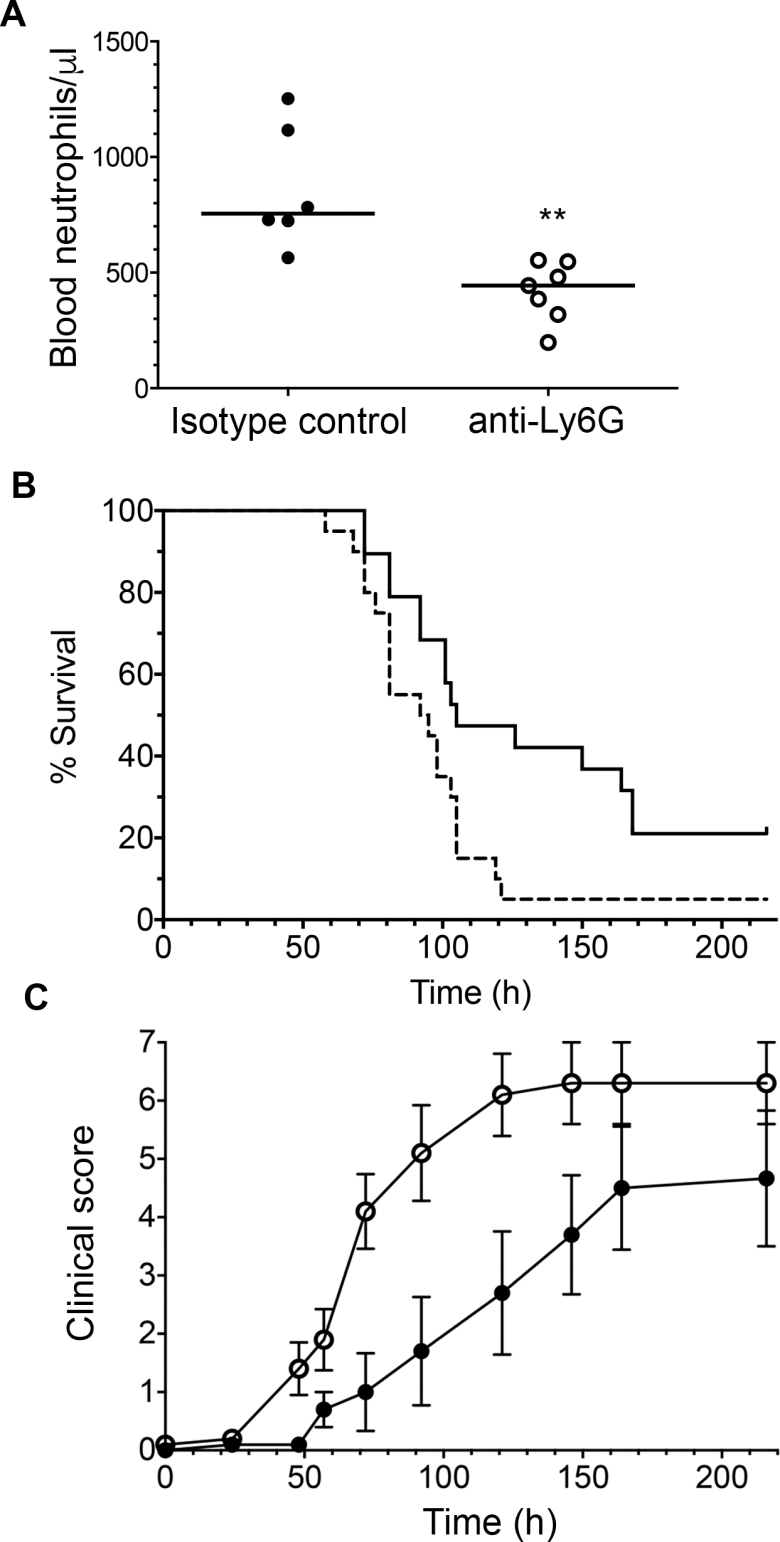
Infection with SRL1 in *Il17ra* KO mice following depletion of neutrophils. *Il17ra* KO mice were infected with 10^6^ cfu/mouse of SRL1by intranasal inoculation 24 hours after intraperitoneal inoculation of 500 μg antibody 1A8 (anti-Ly6G) or isotype matched control and observed for the course of the infection. (A) peripheral blood neutrophil counts following treatment with isotype control (filled circles) or anti-Ly6G (open circles) at 24 hours following infection. Each point is from an individual animal; line indicates median. Difference is significant (**, p < 0.01, Mann Whitney test) (B). Kaplan Meir plot of survival of infected mice. Isotype control treated mice (solid line) had improved survival compared to neutrophil depleted mice (dashed line) (p = 0.017, log rank test). (C) clinical score between isotype treated mice (solid circles) and neutrophil depleted mice (open circles) Differences are significant between the groups (p < 0.0001, 2-way ANOVA).

Improvement in outcome of neutrophil-depleted WT mice after SRL1 infection shows that neutrophils mediate adverse effects after infection with this highly capsulated strain. IL-17 induces neutrophil recruitment and activation; this may thus account for the improved outcome in *Il17ra* KO mice. However, IL-17 also induces other effects, such as production of antimicrobial peptides and synergy with other inflammatory cytokines. In order to determine the relative importance of these IL-17-mediated neutrophil and non-neutrophil effects, we examined the effects of neutrophil depletion in *Il17ra* KO animals infected with SRL1. As before, anti-Ly6G antibody treatment reduced peripheral blood neutrophils (Fig 7A). In contrast to results in WT mice, in the *Il17ra* KO animals, outcome and clinical scores were significantly worse in the neutrophil depleted group (Fig 7B and 7C). These results are considered further in the Discussion.

### Cellular sources of IL-17 within infected lung

**Fig 8:**
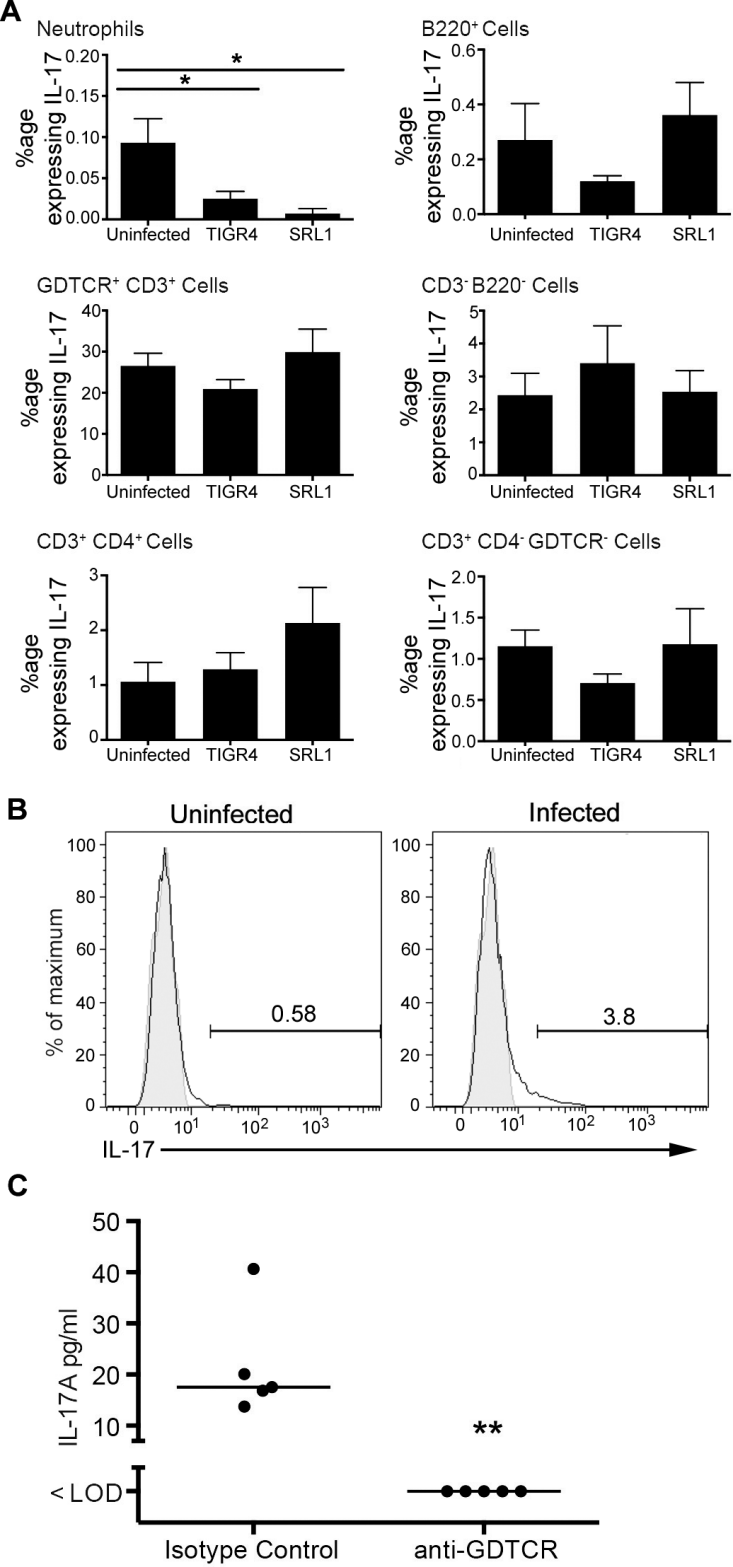
Cellular sources of IL-17A in pneumococcal infection. (A) Proportion of cells staining positive for IL-17 following stimulation with PMA and ionomycin in the presence of Brefeldin 48 hours post infection. Bars are means of 6 determinations; error bars are SEM. Differences between groups were tested by one-way ANOVA and where significant differences from the uninfected group tested by Dunnett’s test. *, p < 0.05, **, p < 0.01. (B) Flow cytometry of *In vivo* expression of IL-17 by gamma delta T lymphocytes from animals treated with Brefeldin A 6 hours prior to infection and culled 6 hours post infection. Shaded area is isotype control staining. Representative of 2 independent experiments. (C) BALF IL-17A concentration in animals treated with a gamma delta T cell depleting antibody (anti-GDTCR) or isotype control 24 hours prior to infection and culled 12 hours after infection. Each symbol is an individual animal; line is the median. Difference between the groups is significant (Mann Whitney test, p = 0.008).

We wished to ascertain the cellular origins of IL-17 in the lung following pneumococcal infection, and to determine whether there were any differences between the pneumococcal strains. We used flow cytometric analysis of cells from lung homogenates stained with a panel of antibodies to identify a variety of cell lineages as set out in the Methods. Cells from control, uninfected animals were analyzed together with those from animals 48 hours after infection with TIGR4 and SRL1. We found that because the inflammatory infiltrate had a patchy localization within infected lungs, determination of absolute numbers of the different cell types within the lung varied very considerably between different experimental animals. However, we were able to compare the relative proportion of each cell type before and after infection that could produce IL-17 after *ex vivo* stimulation with PMA and ionomycin and staining for intracellular Il-17A (Fig 8A). This determination was not influenced by differential recovery of inflammatory cells between animals. The cell type most likely to produce IL-17A following stimulation was γδ TCR^+^ cells, but there was no change between infected and uninfected mice. The only significant change noted was a reduction in the proportion of neutrophils capable of synthesizing intracellular IL-17 following infection, although the absolute numbers of these cells are very low. As these cells are stimulated to produce IL-17 *ex vivo*, it does not necessarily reflect their activity during an infection. To address this question, we administered brefeldin A to infected animals prior to harvesting lung cells to allow accumulation of IL-17 in those cells actively producing this cytokine during infection. Cell homogenates were then analyzed for intracellular IL-17 production without further stimulation. The only cell lineage that produced IL-17A under these conditions were γδ T cells, showing a rise in intracellular IL17A^+^ cells from 0.58% to 3.8% following infection (Fig 8B). To confirm that γδ T cells were a significant source of IL-17A during infection, we administered an antibody that depletes γδ T cells 6 hours before infection. We then assayed IL-17A in BALF 6 hours after infection in these animals and those that received an isotype control (Fig 8C). IL-17A production in BALF was largely abrogated in animals in which γδ T cells were depleted compared to the isotype controls. Thus, the majority of IL-17 produced locally within the lung following pneumococcal infection in this model originates from γδ T cells.

### Role of IL-17 in nasopharyngeal colonization of mice with TIGR4 and SRL1

**Fig 9:**
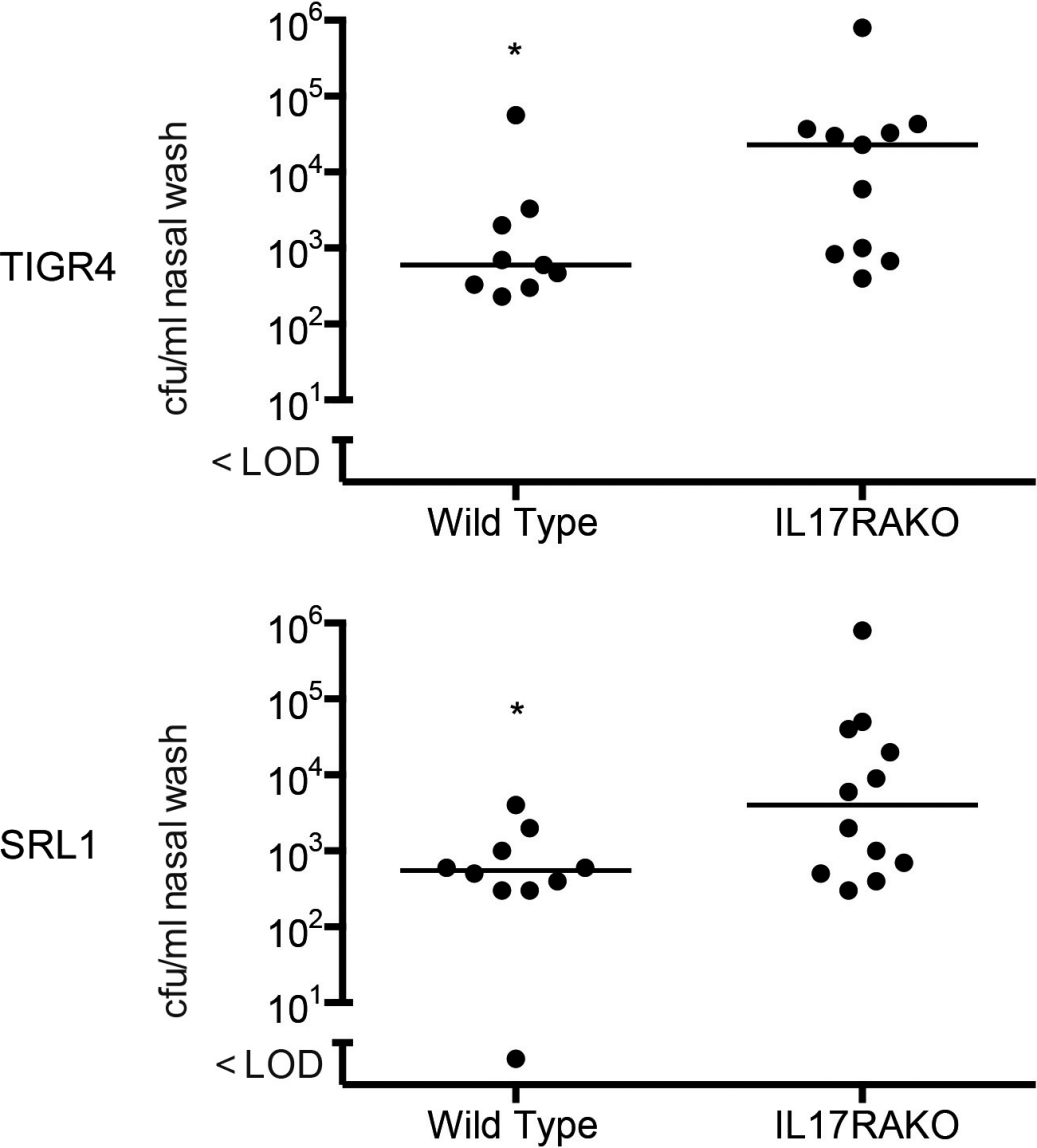
Nasal colonization with TIGR4 or SRL1 in IL-17RA^-/-^ mice. Bacterial counts in nasal wash enumerated 24 after inoculation. Line shows median value; differences are significant by Mann Whitney test, *, p < 0.05.

We examined the effect of IL-17 on nasopharyngeal colonization by TIGR4 and SRL1. 7 days after inoculation of these strains into the anterior nares, there were significantly higher numbers of bacteria recovered from the nasopharynx of *IL17ra* KO mice compared to WT mice for both the TIGR4 and SRL1 strains (Fig 9). Thus, the deleterious role of IL-17 following pulmonary infection with SRL1 is not seen with nasopharyngeal colonization, where IL-17 is protective for both TIGR4 and SRL1.

### Role of IL-17 in host defence against pulmonary infection with an additional highly encapsulated non-invasive pneumococcal strain SRL2 (serotype 6B)

Although capsular phenotype is a major determinant of outcome following infection, other factors could contribute to the observed differences between TIGR4 and SRL1. We thus explored the effects of infection of a TIGR4 capsule switched mutant that had been engineered to express capsule type 3, the capsular type of SRL1 [36]. We found this strain was avirulent when given by nasal inoculum in doses up to 1 x 10^7^ per mouse, and could not be recovered from blood following intraperitoneal inoculation of mice. Loss of virulence of capsule switched mutants of the pneumococcus has been documented previously [37–39].

To explore further the link between capsule type and IL-17 mediated host immunity, we determined whether another highly encapsulated non-invasive strain would also show a survival benefit following infections in *Il17ra*KO mice. We selected another train, SRL2, which was a serotype 6B capsular phenotype, known to be a relatively non-invasive strain with a large capsule [9]. We confirmed the relatively large size of the capsule compared to TIGR4 using the same methods as in Fig 1.

**Fig 10:**
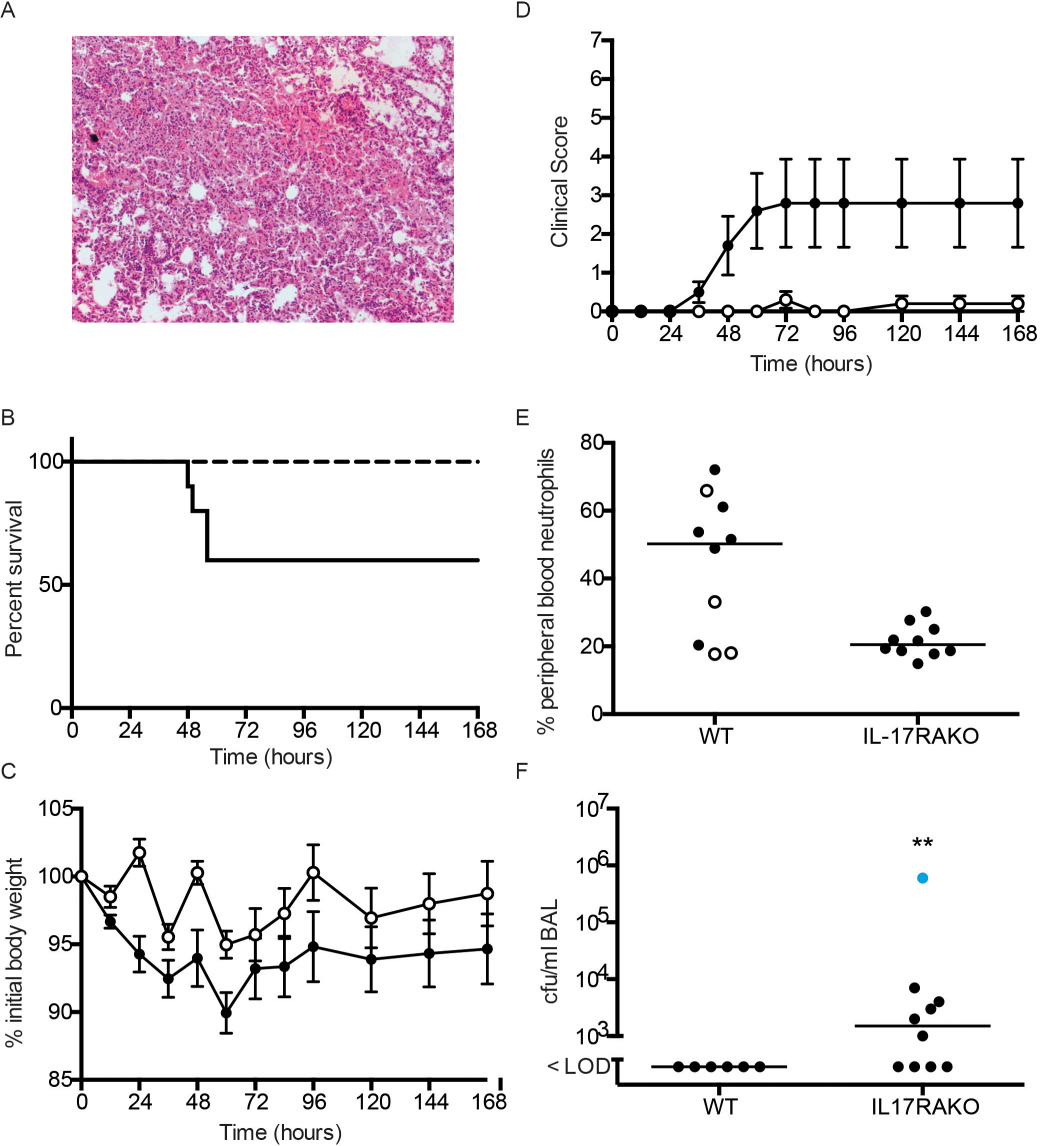
Infection of IL-17RA^-/-^ mice with SRL2. (A) Representative area of inflamed lung from H&E stained lung sections of mice infected with SRL2. (B) Kaplin-Meier plot of survival following infection of wild type (solid line) or IL-17RA^-/-^ (dashed line) mice with SRL2. Survival of IL-17RA^-/-^ mice was improved (logrank test, p = 0.03). (C) Weight following infection in IL-17RA^-/-^ mice (open circles) and control mice (closed circles). Points are means (n = 10); error bars are SEM. Differences between the groups were not significant (two-way ANOVA, p = 0.06). (D) as (C) but showing clinical score. Difference between the groups was significant (two-way ANOVA, p < 0.001). (E) peripheral blood neutrophil percentage in WT and IL-17RA^-/-^ mice 24 h after infection. Line shows median value. Closed symbols are animals that survived to the end of the 7 day experiment. Differences between the groups are significant (Mann Whitney test, p = 0.03). (F) BALF bacterial counts from mice that survived infection with SRL2. Line shows median value. Differences between the groups are significant (Mann Whitney test, p = 0.01). Animal shown in blue had mild clinical illness; the remainder had no clinical signs of infection.

Infection with SRL2 produced an extensive pneumonia with large areas of confluent consolidation as seen with infection with SRL1 (Fig 10A). There was a clear and significant difference in survival between WT and *Il17ra* KO animals, with none of the *Il17ra* KO animals dying following infection, compared to a 40% mortality in the WT animals (Fig 10B). There was a trend towards decreased weight loss in the *Il17ra* KO animals following SRL2 infection (Fig 10C) and significantly lower clinical scores in this group compared to the infected wild type animals (Fig 10D). As observed with the other pneumococcal infections, blood neutrophils were significantly reduced in the *Il17ra* KO animals after infection (Fig 10E). In the surviving animals 7 days after infection, all of whom showed no evidence of any ongoing infection, we found 60% of the *Il17ra*KO mice had detectable levels of SRL2 in BALF, a similar observation to the surviving *Il17ra* KO mice following SRL1 infection.

## Discussion

We have shown here that protective versus harmful effects of IL-17 in the innate immune response to acute pneumococcal infection in a murine model are critically dependent on pneumococcal strain. IL-17 is protective against pulmonary infection with TIGR4, a representative invasive strain of relatively low capsular thickness, but has an adverse effect on outcome following infection with 2 separate highly encapsulated strains (SRL1 and SRL2) that are representative of strains of lower invasive potential. Since neutrophil depletion improved survival following infection with these strains, we believe that the deleterious effect of IL-17 against these strains in this model is mediated by neutrophils. This is also supported by the interactions of purified neutrophils with TIGR4 and SRL1, which show that the highly encapsulated SRL1 strain is better able to avoid neutrophil uptake and killing compared to TIGR4. Neutrophil-mediated damage has been implicated as a key pathogenic factor in adult respiratory distress syndrome [40] and contributing to tissue damage by bacterial and nematode infection [41, 42]. Neutrophil depletion enhanced survival in a model of pneumococcal infection caused by a non-hemolytic serotype 8 strain [43]. We propose that extensive neutrophil accumulation as seen in Fig 4, mediates enhanced tissue damage following pulmonary infection with highly encapsulated pneumococcal strains, allowing enhanced growth and passage of bacteria out of the lung and increasing mortality.

Capsular phenotype is an important determinant of many different aspects of pneumococcal pathogenicity. Here, we have developed a murine model of pneumonia that reflects the clinical course of pneumococcal infection by different serotypes. TIGR4 (serotype 4) represents an ‘invasive’ strain that is less commonly found as a colonizing strain compared to its isolation as an invasive organism, that typically would imply isolation from blood or csf. SRL1 is a representative relatively “non-invasive” strain, with opposite properties. In our model, TIGR4 produces a patchy bronchopneumonia but transits rapidly through the lung to invade blood. SRL1 infection results in much more extensive lung consolidation and only invades blood relatively late. Most human studies of pneumococcal infection rely on definitive diagnosis by blood culture and will only identify a minority of pneumonias caused by the pneumococcus. However, the differences in the phenotype of infection produced by these strains in the model described here is certainly consistent with available data from human studies.

Factors other than capsule will impact on pathogenicity of different pneumococcal strains. We attempted to test the effect of capsular phenotype alone, by examining the outcome of infection with a TIGR4 capsule switch mutant expressing the type 3 capsule. However, this strain was not virulent in our model. This lack of virulence in capsule switch mutants has been described in previous reports [37–39]. These studies suggest that capsular type imposes restrictions on expression or accessibility of key virulence determinants, such that changing capsular type may attenuate the virulence of a specific pneumococcal strain. Co-evolution of capsule and virulence determinants thus ensures optimal expression of these determinants in a specific capsule type. Direct comparison of infection with SRL1 and 2, both highly encapsulated strains, shows outcome is improved in the absence of IL-17 signaling, in contrast to the results seen with TIGR4, a strain with relatively thin capsule. We also show that neutrophil depletion attenuates the effects of infection with the type 3 capsular expressing strain and thus propose that IL-17 exerts a deleterious effect in infection with SRL1 and SRL2 because of IL-17-mediated neutrophil damage. Other factors might contribute to the observed difference in the effect of IL-17 on infection with these different strains, but our data are consistent with a hypothesis that the thick capsular phenotype leads to IL-17dependent neutrophil accumulation in the lung, with consequent neutrophil-mediated lung damage and escape of the microbe into the systemic circulation.

There is, however, a balance between the beneficial effects of neutrophils in bacterial clearance and potential deleterious effects when present in excess. This is demonstrated in our experiments analysing the effects of neutrophil depletion in *Il17ra* KO mice. Compared to the effects on neutrophil depletion in WT mice, in mice lacking IL-17 signalling, neutrophil depletion worsened outcome. We propose that the most likely explanation of these results is that there is a critical level of neutrophils below which effective bacterial clearance is severely compromised. Thus, in the *Il17ra* KO animals, neutrophil recruitment and activation is attenuated to an extent that ameliorates pulmonary disease, but prevents bacterial escape into the systemic circulation and subsequent severe disease. When neutrophil levels are depleted further in the *Il17ra* KO animals, they are no longer able to restrict the infection and animals thus develop severe disease and die. An alternative view would be that non-neutrophil effects of IL-17 account for worse outcome in SRL1 infection. We do not favour this interpretation, as it would be difficult to understand how IL-17 effects such as induction of antimicrobial peptides could worsen outcome, although we cannot totally exclude a role for non-neutrophil effects of IL-17 on outcome in SRL1 and 2 infection.

BALF pneumococcal bacterial counts were about 2 log higher in WT SRL1 infected animals compared to TIGR4, in keeping with a view that the larger type 3 capsule favours retention within the lung and hence the ‘non-invasiveness’ of this serotype in human studies. This is in comparison to TIGR4, a representative of an ‘invasive’ human phenotype with thinner capsule. In SRL1 infection, lack of IL-17 effects resulted in lower transit of the microbe into blood but without a concomitant rise in pneumococcal bacterial counts in BALF. We propose that this reflects the ability of non-IL-17 innate immune mechanisms to keep SRL1 infection within the lung in check. This is supported by the observation that in the absence of IL-17 RA, significant numbers of viable SRL1 and SRL2 bacteria could be recovered from lungs of completely healthy mice. This suggests that innate mechanisms other than those driven by IL-17 can keep SRL1 and SRL2 infection controlled but not eradicated within the lung. In contrast, no animals that survived TIGR4 infection had evidence of viable bacteria remaining within their lungs. The large capsules of SRL1 and SRL2 thus would appear to favour retention of the organism within the lung, which in the absence of IL-17 mediated killing results in viable bacteria remaining but not causing further disease. Possible mechanisms for this retention of highly encapsulated strains could be an inability to traverse the alveolar epithelial barrier or retention by NETs as shown in this study. Further work will be required to define the exact mechanisms involved.

IL-17 mediated protection against pneumococcal colonization has been demonstrated in a number of studies [25, 31]. Thus, a robust pneumococcal-specific IL-17 response following vaccination with a whole-cell or recombinant pneumococcal vaccine has been proposed to be beneficial in providing host defence [17, 23]. However, while IL-17 is instrumental in preventing nasopharyngeal colonization, the role in disease states is less clear. There is strong evidence that antibody mediated immunity is the dominant protective mediator in bacteremia [44]. In our study, IL-17 reduced the likelihood of bacteremia in TIGR4 infection but did not affect disease progression in bacteremic animals. The role of IL-17 in pulmonary infection was recently explored in the context of acquired immunity [31]. IL-17 accelerated lower respiratory tract clearance of a serotype 19F strain of relatively low virulence in a manner that was partially dependent on neutrophils. Our data support this finding that IL-17 is required to efficiently clear highly encapsulated pneumococci from the lower airways. However, in severe infection the deleterious effect of neutrophil recruitment outweighs benefit and worsens outcome. Thus, the data presented here strongly suggest that, under some circumstances, disease from highly encapsulated strains will be exacerbated by IL-17. How far these results might be applicable to the role of IL17 in human pneumococcal pneumonia is not clear. Rare families with absence of IL17RA or other elements of the IL17 axis have been described. The main infectious complication experienced is that of chronic mucocutaneous candidiasis, but in a study of 21 patients with inherited *IL-17RA* deficiency, 3 experienced recurrent lobar pneumonia [45]. Given that the pneumococcus is the commonest cause of lobar community-acquired pneumonia, this suggests that IL-17RA is key in human host defence against pneumococcal disease. However, not enough data exist to draw any conclusions about the effects of different capsular types on the outcome of pneumococcal infection in such patients.

## Materials and methods

### Bacterial strains

TIGR4 (Serotype 4) is a clinical isolate that has been previously described [46]. SRL1 (Serotype 3) and SRL2 (Serotype 6B) were obtained from the Scottish Haemophilus, Legionella, Meningococcus and Pneumococcus Reference Laboratory. The TIGR4 capsular switch mutant to type 3 was kindly provided by Professor Jerry Brown, University College London. Quantification of bacteria was performed by serial dilution onto 5% horse blood agar plates.

### Animals and models of colonization and disease

All mice used were on a C57Bl/6 background. I*Il17ra* KO mice were obtained from Jay Kolls (University of Pittsburgh). Mice were housed in pathogen free conditions at the University of Glasgow and maintained according to standard procedures. All research was conducted in accordance with institutional guidelines and in accordance with Home Office regulations. Models of pulmonary infection [47] and colonization [48] were performed as previously described. Briefly, bacterial stocks were prepared by passage through mice and kept frozen for use in infection models. Mice were inoculated via the intra-nasal route with light gaseous anaesthesia in models of pulmonary infection and gentle immobilization in the absence of anaesthesia for colonization models. Mice were monitored using a standardized scoring system and culled either at a pre-determined time point or if the severity score reached the specified level. In some experiments, mice were administered the monoclonal antibodies 1A8 [35] (mouse neutrophil specific antibody to Ly6G; 500 μg, Bio-X-cell), UC7-13D5 [49] (mouse γδ cell specific antibody to murine γδ T cell receptor; 100 μg, eBioscience) or appropriately matched isotype control by intraperitoneal injection 24 hours prior to the start of the experiment. In other experiments, mice were administered 250 μg Brefeldin A by intraperitoneal injection 6 hours prior to being culled.

Blood was obtained by tail vein puncture. Bronchial and pleural lavage were performed as previously described. Neutrophil counts from these fluids were obtained by lysing red blood cells and staining neutrophils with anti-Ly6G (RB6-8C5, PE or eFluor605NC, eBioscience) before gating on side scatter^+^Ly6G^+^. IL-17A ELISA was conducted using the Ready,Set,Go kit (eBioscience) according to the manufacturer’s instructions.

Lung myeloperoxidase activity was quantified by placing a lobe of lung in 50 mMol KH2PO4 with 0.5% hexadecyltrimethylammonium bromide in ddH2O, homogenizing with a tissue grinder and lysing by sonication. MPO activity was then determined as previously described [50]. To determine the wet/dry ratio, a single lobe of lung was blotted on tissue paper to remove surface fluid and carefully weighed. The lung was then placed in an oven at 60°C for 48 hours and the desiccated lung weighed again.

Lung homogenates were prepared by perfusing the lungs with ice cold saline via the right ventricle before removing the lungs and cutting them into small pieces. Homogenisation was performed enzymatically in IMDM media supplemented with 1% foetal bovine serum, 50 μg/ml DNAse and 0.65 units/ml liberase TM (Roche, CH) followed by passing fragments through a 40 μm cell strainer. After lysing red cells and washing, cells were prepared for flow cytometry according to the manufacturer’s instructions. Where indicated, cells were incubated with media containing phorbol 12-myristate 13-acetate (PMA) (50 ng/ml), ionomycin (1 μg/ml) and Brefeldin A (5 μg/ml). Cells were then incubated for 6 hours at 37°C. Cells were stained with FVD 750 (eBioscience) before antibody staining. Antibodies used during flow cytometry were: anti-Ly6G (RB6-8C5, PE or eFluor605NC, eBioscience), anti-B220 (RA3-6B2, PE-Cy7, Biolegend), anti-CD3 (17A2, PE-Cy7, Biolegend), anti-CD4 (RM4-5, PerCP-Cy5.5 or eFluor605NC, eBioscience) and anti-γδTCR (UC7-13D5, A. fluor 488, Biolegend). Anti-IL-17A (TC11-18H10.1, PE, eBioscience) was used following fixation and permeabilization according to the manufacturer’s instructions. Cells were re-suspended in a buffer containing PBS supplemented with 2mmol/l EDTA, 10% foetal calf serum (FCS) and 0.09g/l sodium azide and analyzed on an ADP-CyAN flow cytometer (Beckman Coulter) or LSR II (BD Biosciences).

For neutrophil immunohistochemistry lungs were fixed in zinc salt fixative [51] for 48 hours before tissue processing into paraffin blocks. After cutting and rehydration, endogenous peroxidase was blocked by immersion in a 3% H2O2 in PBS for 30 minutes and endogenous biotin blocked using an Avidin/Biotin blocking kit (Vector Laboratories) according to the manufacturer’s instructions. After blocking and permeabilization, sections were incubated overnight at 4°C with 500 ng/ml anti-Ly-6G (1A8, eBioscience) or matched isotype control. The following day, sections were washed before incubation for 1 hour at room temperature with secondary antibody and subsequently washed again. Sections were then incubated for 30 minutes at room temperature with avidin/biotinylated peroxidase complex (VECTASTAIN ABC, Vector Laboratories) and washed. Sections were then incubated at room temperature with 3,3’-diaminobenzidine (DAB, Vector Laboratories) until adequate colour change occurred. Sections were rinsed in water before nuclei were stained for 5 minutes with methyl green solution and rinsed in distilled water. Sections were then dehydrated rapidly through serial alcohols, cleared in two changes of xylene for 5 minutes each and then analyzed.

### RT-PCR

Immediately after dissection, lungs were placed in RNA later solution (Life Technologies) and frozen at -80°C until use. After gentle thawing, lungs were transferred into a 2ml centrifuge tube with a 5mm steel bearing (Qiagen) and 1ml Trizol (Life Technologies). Samples were loaded into a TissueLyser LT (Qiagen) and pulsed at 50 Hz for 5 minutes. RNA was then extracted from the lung homogenate using a PureLink RNA Mini Kit (Life Technologies) and first strand cDNA generated using a SuperScript First-Strand Synthesis System (Life Technologies) using oligoDT primers according to the manufacturer’s instructions. PCR was then conducted using Fast SYBR Master Mix (Life Technologies) according to the manufacturer’s instructions and specific primers (sequence available on request). Expression was normalized to expression of the TATA-binding protein.

### Capsular thickness

Capsular thickness was determined by Alexa fluor 488 dextran exclusion microscopy as previously described [52]. Transmission electron microscopy was performed using ruthenium red to preserve the capsule as previously described [53].

### Neutrophil phagocytosis, killing and NET formation

Mouse neutrophils were obtained by induction of peritonitis by injection of 9% Casein [54]. Human neutrophils were obtained from whole blood by density centrifugation as previously described [55]. Phagocytosis and killing studies were performed as previously described [56]. Additionally, neutrophil phagocytosis was quantified by flow cytometry and confocal microscopy by incubating neutrophils with bacteria stained with 10μ eFluor 450 Proliferation Dye.

To assess NET formation, human neutrophils were seeded in RPMI 1640 medium supplemented with 2% heat inactivated human serum onto sterile, round glass coverslips placed in 24 well tissue culture plates. Neutrophils were infected with bacterial strains stained with eFluor 450 as previously described or activated with 1μg/ml PMA as a positive control and incubated at 37°C under 5% CO2 for 4 hours. Neutrophils were fixed in 4% paraformaldehyde in PBS for 60 minutes before being removed from tissue culture plates with curved forceps and washed three times by inversion onto a drop of PBS on a parafilm sheet. Neutrophils were then permeabilised, blocked and stained with anti-neutrophil elastase (rabbit polyclonal IgG, AbCam) and SYTOX Orange (ThermoFisher) as described above before coverslipping and mounting with Fluoromount- G (eBioscience) and imaging with confocal microscopy.

### Ethics statement

All animal experiments were carried out in strict accordance with UK Government Home Office Regulations concerning animal experimentation under Project Licence PPL 60/4631 as well as the Animal Experiments Review Board of the University of Glasgow. These regulations adhere to the European Union regulations concerning the use of animals in scientific research under Directive 2010/63/EU of the European Parliament as well as the Animals (Scientific Procedures) Act 1986 of the UK Parliament.

### Statistics

Between group analyses were performed using t tests, one or two way ANOVA, Kruskal Wallis test or Mann Whitney test as indicated. Survival curves were compared using the logrank test. P values < 0.05 were taken as significant.

## Supporting information

S1 Fig Gene expression of known IL-17 target genes. Expression was assessed using qRT-PCR of cDNA derived from lung RNA extract (n = 3/group). Columns are means of expression levels relative to wild type control animals; error bars are sem. Significance levels by two sample t test are shown: (*: P < 0.05, **: P < 0.01, ***: P < 0.001).
(TIF)

S2 Fig Change in body weight 24 hours after infection with TIGR4. Each point is an individual animal; line shows median. Differences between the medians were assessed by Mann Whitney test (** p < 0.01).
(TIF)

S3 Fig Lung wet/dry mass ratio 24 hours after infection with TIGR4. Each point is an individual animal; line shows median. Differences between the medians were assessed by Mann Whitney test (ns = not significant).
(TIF)
