# Supplementary figures and images for: IL-17 can be protective or deleterious in murine pneumococcal pneumonia
Source: PLoS Pathog. 2018 May 29;14(5):e1007099. doi: 10.1371/journal.ppat.1007099 (PMC5993294; doi:10.1371/journal.ppat.1007099)

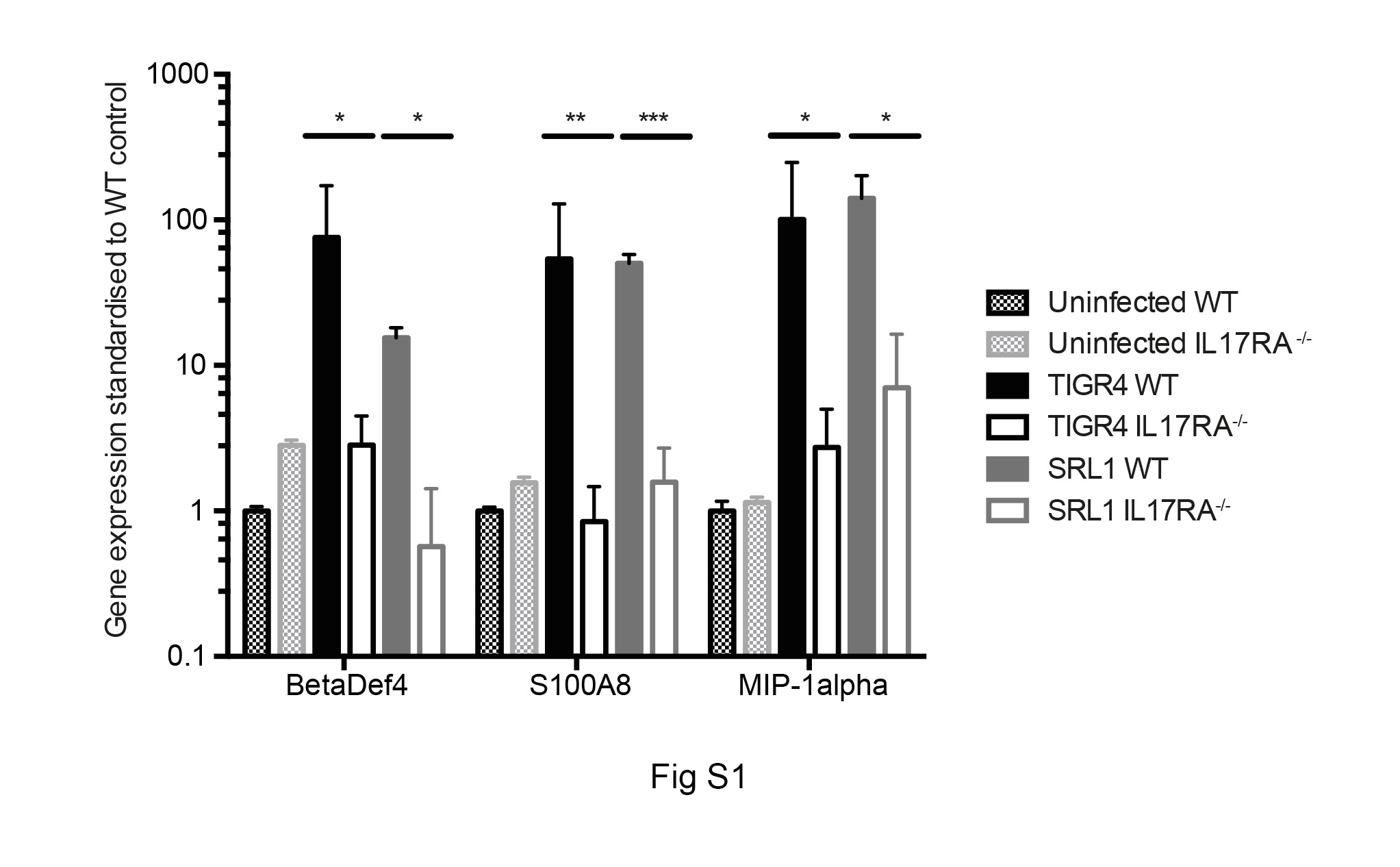

Supplement: S1 Fig — Expression was assessed using qRT-PCR of cDNA derived from lung RNA extract (n = 3/group). Columns are means of expression levels relative to wild type control animals; error bars are sem. Significance levels by two sample t test are shown: (*: P < 0.05, **: P < 0.01, ***: P < 0.001). (TIF) [file ppat.1007099.s001.tif]

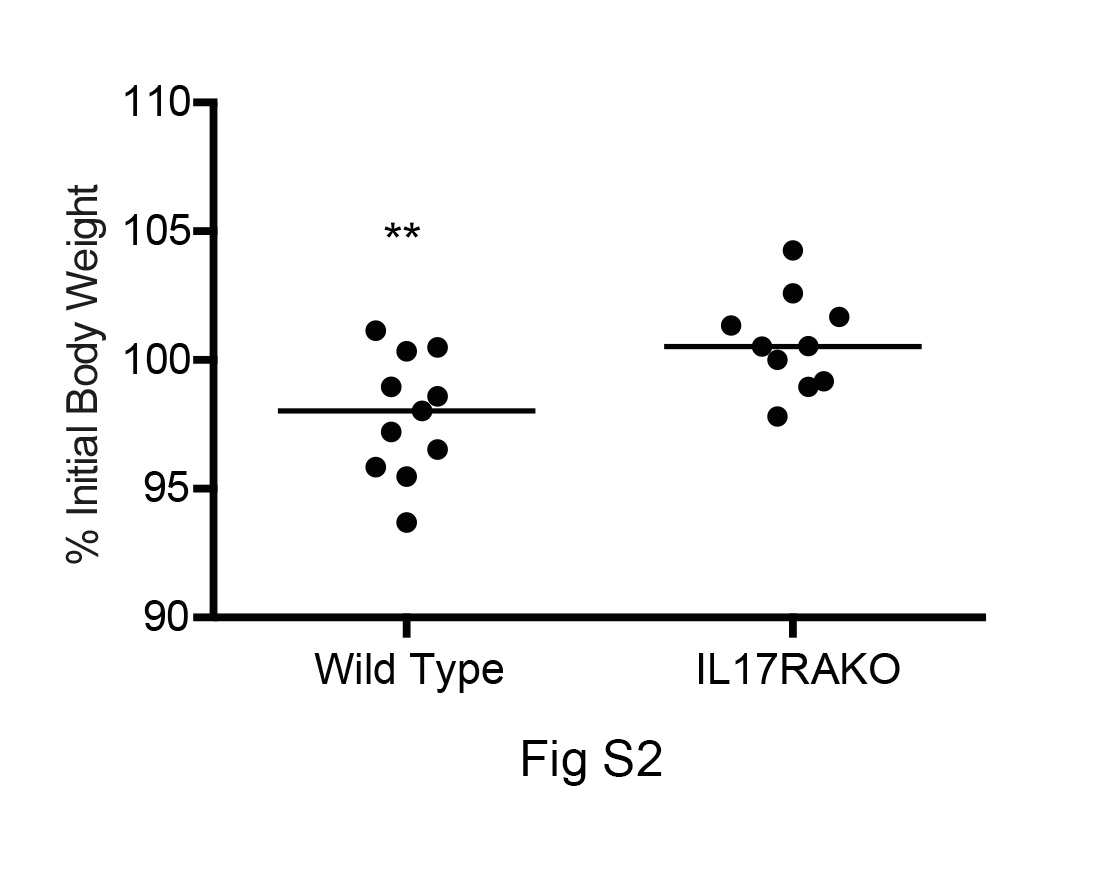

Supplement: S2 Fig — Each point is an individual animal; line shows median. Differences between the medians were assessed by Mann Whitney test (** p < 0.01). (TIF) [file ppat.1007099.s002.tif]

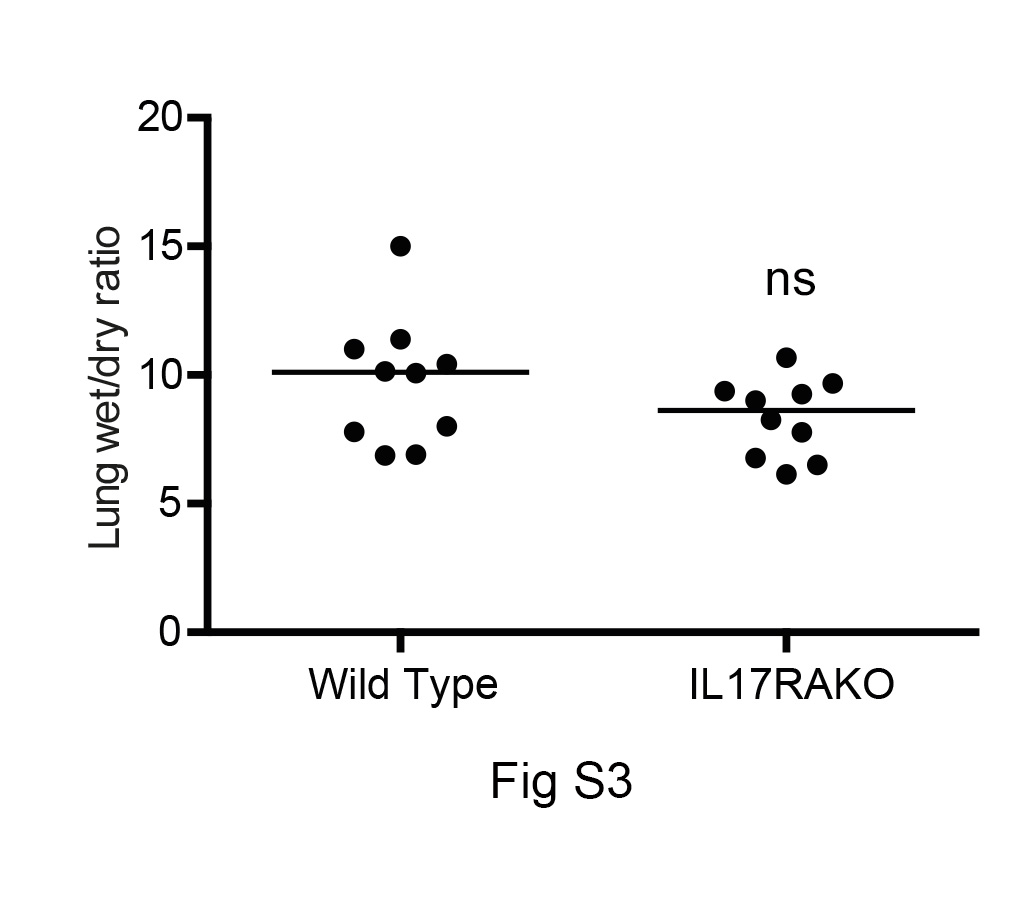

Supplement: S3 Fig — Each point is an individual animal; line shows median. Differences between the medians were assessed by Mann Whitney test (ns = not significant). (TIF) [file ppat.1007099.s003.tif]
